# Enhancing Esthetics Using Prosthetic Cheek Plumper With Magnet Retention for Rehabilitation of an Edentulous Patient: A Case Report

**DOI:** 10.7759/cureus.50013

**Published:** 2023-12-05

**Authors:** Seema Sathe, Rajanikanth K, Anjali Borle, Tanvi Jaiswal

**Affiliations:** 1 Prosthodontics, Sharad Pawar Dental College and Hospital, Datta Meghe Institute of Higher Education and Research, Wardha, IND; 2 Dentistry, Sharad Pawar Dental College and Hospital, Datta Meghe Institute of Higher Education and Research, Wardha, IND

**Keywords:** edentulous patient, complete denture, sunken cheeks, magnetic attachment, cheek plumper

## Abstract

The patient's state of health is significantly impacted by aesthetics. The face undergoes various changes as we age, including the loss of fat and muscle tonicity. The loss of teeth and these modifications accentuate the look of sunken cheeks. As a result, facial deformity may have an effect on a person's mental health, especially young people who are edentulous patients. Cheek plumpers and complete dentures (CDs) can be utilized to help these people recover or enhance aesthetics. The manufacturing of a CD with magnets is described in this case report.

## Introduction

Denture aesthetics is defined as the aesthetic impact that a dental prosthesis has on a person's dignity, character, attractiveness, and beauty [1]. Complete tooth loss in elderly people results in the destruction of the residual ridge and decreased muscular tone, causing cheeks to sink, causing a scooped-out, collapsed look, and amplification of skin folds due to tissue laxness. This aged look frequently has an impact on the patient's self-perception, which results in emotions of social rejection, psychological pressures, and more age-concealing treatments [2]. Cheeks have a significant impact on face appearance. A person with collapsed or depressed cheeks may appear older. The prosthesis's flanges can occasionally be sufficient to impart fullness to the lips and cheeks, but this is not always sufficient [3]. While traditional complete dentures (CDs) provide some support for the lips and cheeks, they may be insufficient in situations of tissue thinning and increased muscle strength. Rehabilitating cheek support in these kinds of patients can result in a good aesthetic alteration and boost their self-confidence.

Additional support in the buccal flange area is necessary to address the issue of sunken cheeks. A cheek plumper (CP) or cheek-uplifting device is a prosthesis that supports and uplifts the cheek to offer the necessary aesthetics that would boost the patient's pride. There are two kinds of CPs: detachable and non-detachable. Undetachable CPs are standard prostheses in one piece with adjuncts on both sides of the buccal surfaces of the denture which were polished. While detachable CPs can be separated from the denture. Magnets or customized additions can help with this [4]. This case scenario details the fabrication of a CD equipped with CPs that are attached using magnets, providing a lightweight yet effective means of supporting sunken cheeks in individuals requiring dental restoration.

## Case presentation

**Figure 1:**
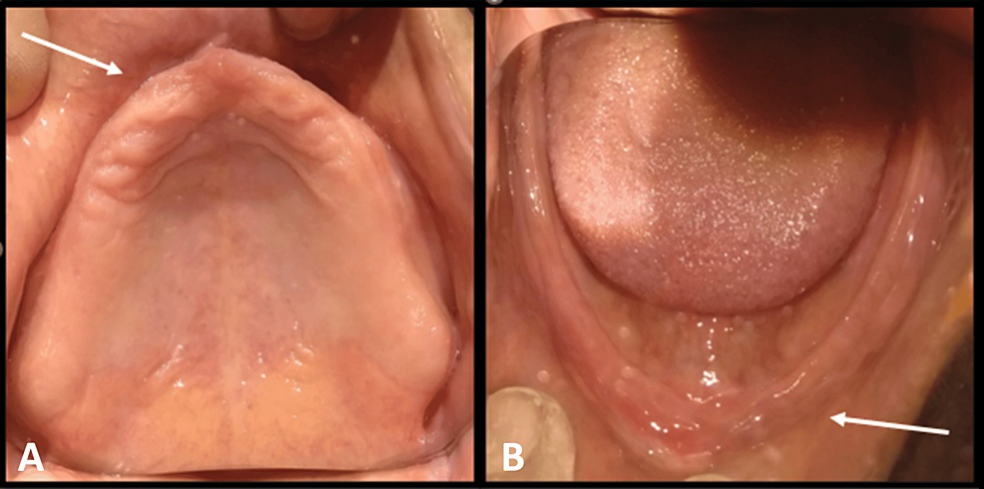
(A) Intra-oral photographs showing maxillary edentulous ridge; (B) Intra-oral photographs showing mandibular edentulous ridge

**Figure 2:**
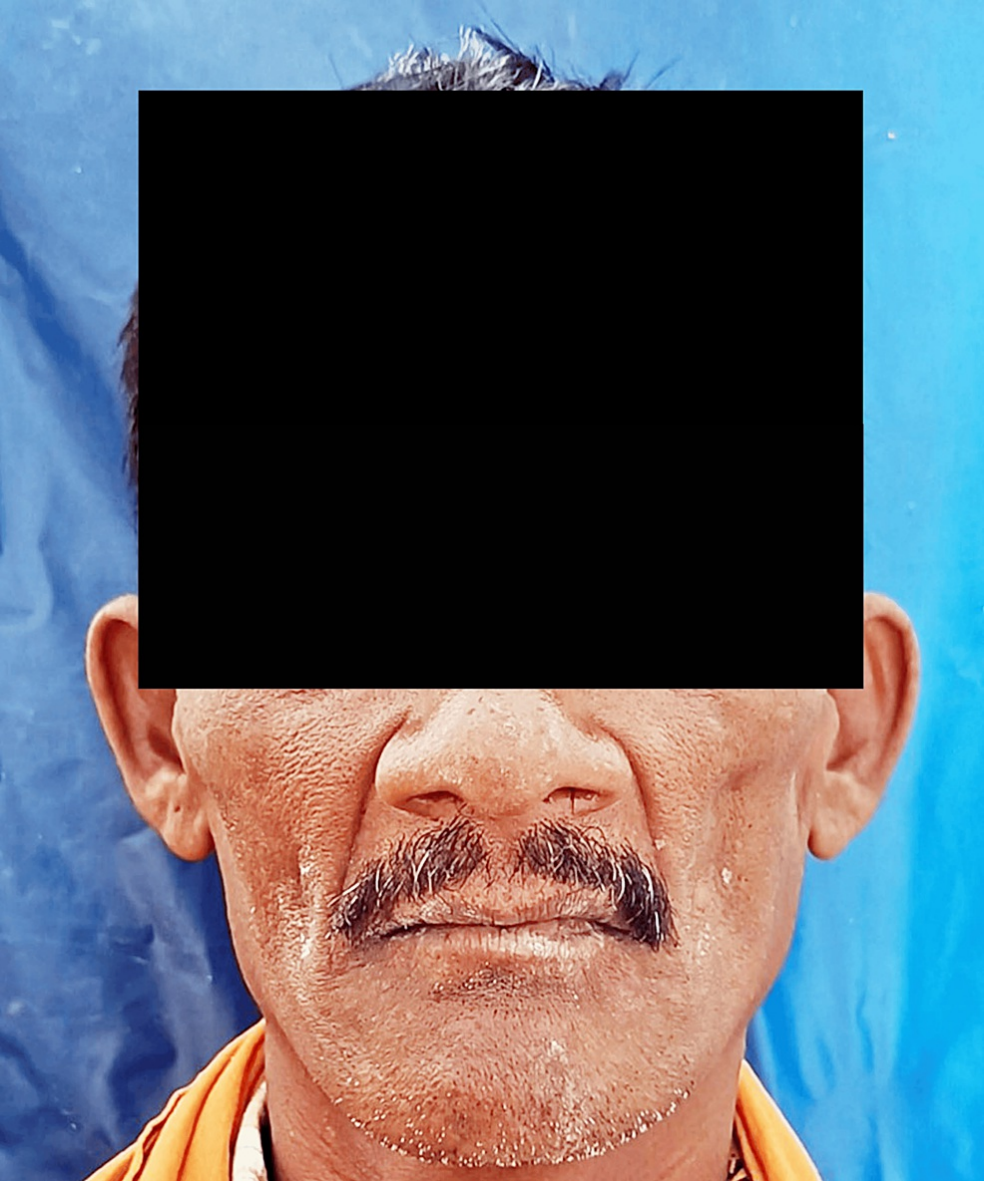
Extra-oral photographs

A 55-year-old male patient arrived at Sharad Pawar Dental College and Hospital, Wardha, seeking dental care for lost teeth and depressed cheeks. Clinical investigation revealed that the patient had no teeth in either the upper or lower dental arches as represented in Figure 1. Extraoral examinations also revealed aged skin and decreased muscle tone, contributing to the cheeks' depressed look as represented in Figure 2.

The patient indicated a great desire for a denture that would improve the appearance of his face. A treatment plan was designed to comply with his dental needs, which included the fabrication of a conventional complete denture as well as the insertion of removable CPs for the maxillary denture. Impression compound (Y- Dents Impression Composition, Medical and Dental Market (MDM) Corporation, Delhi, India) was used to make preliminary impressions of the maxillary and mandibular arches. The unique trays were made with auto-polymerizing acrylic resin. Tissue molding was performed and secondary impressions were made with a zinc oxide eugenol dental impression paste. Jaw relation was recorded.

**Figure 3:**
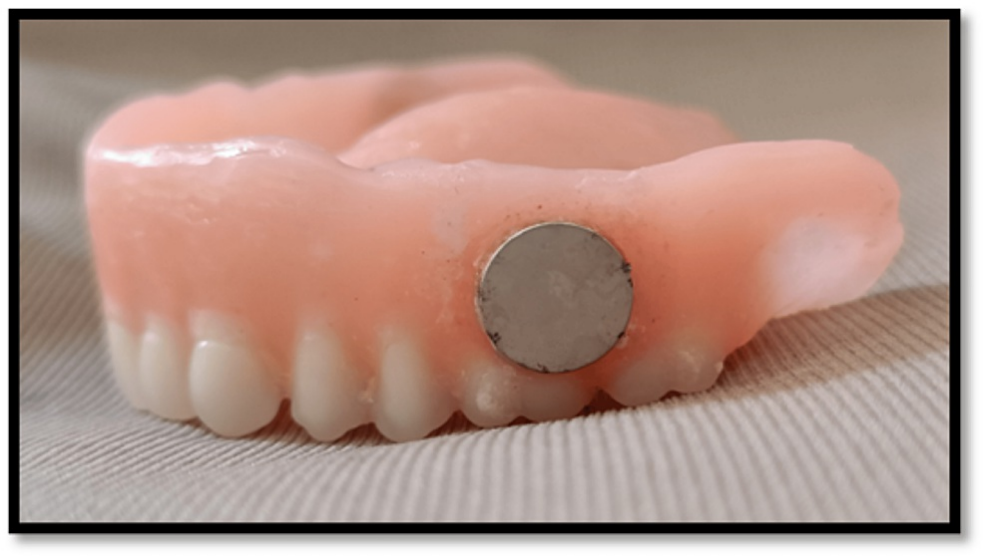
Complete denture with magnetic attachment

**Figure 4:**
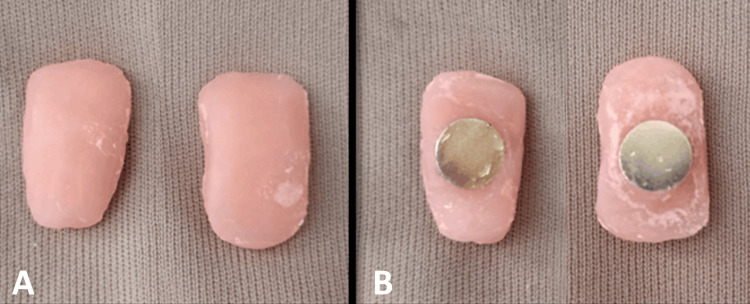
(A) Superior surface of the cheek plumper; (B) Magnetic attachments in cheek plumper

The waxed-up denture was examined for aesthetics and occlusion at the following session. During the same session, wax cheek plumpers were created and magnetized to the maxillary waxed-up denture are simple to install, easy to clean, instantly reseat because of the high magnetic force, and come in small sizes. The buccal flange and CPs of the maxillary denture both had magnets attached to them. The fullness of the cheeks was used to evaluate the patient's appearance. The patient's face looked improved after using the CP. The client was pleased with his physical look. The waxed plumper and magnet were detached from the waxed-up denture. The edges of the magnet were delimited from the wax to ease magnet replacement after dewaxing. These flasking and dewaxing procedures were then performed independently for the final step of prosthesis and CPs as represented in Figures 3, 4.

**Figure 5:**
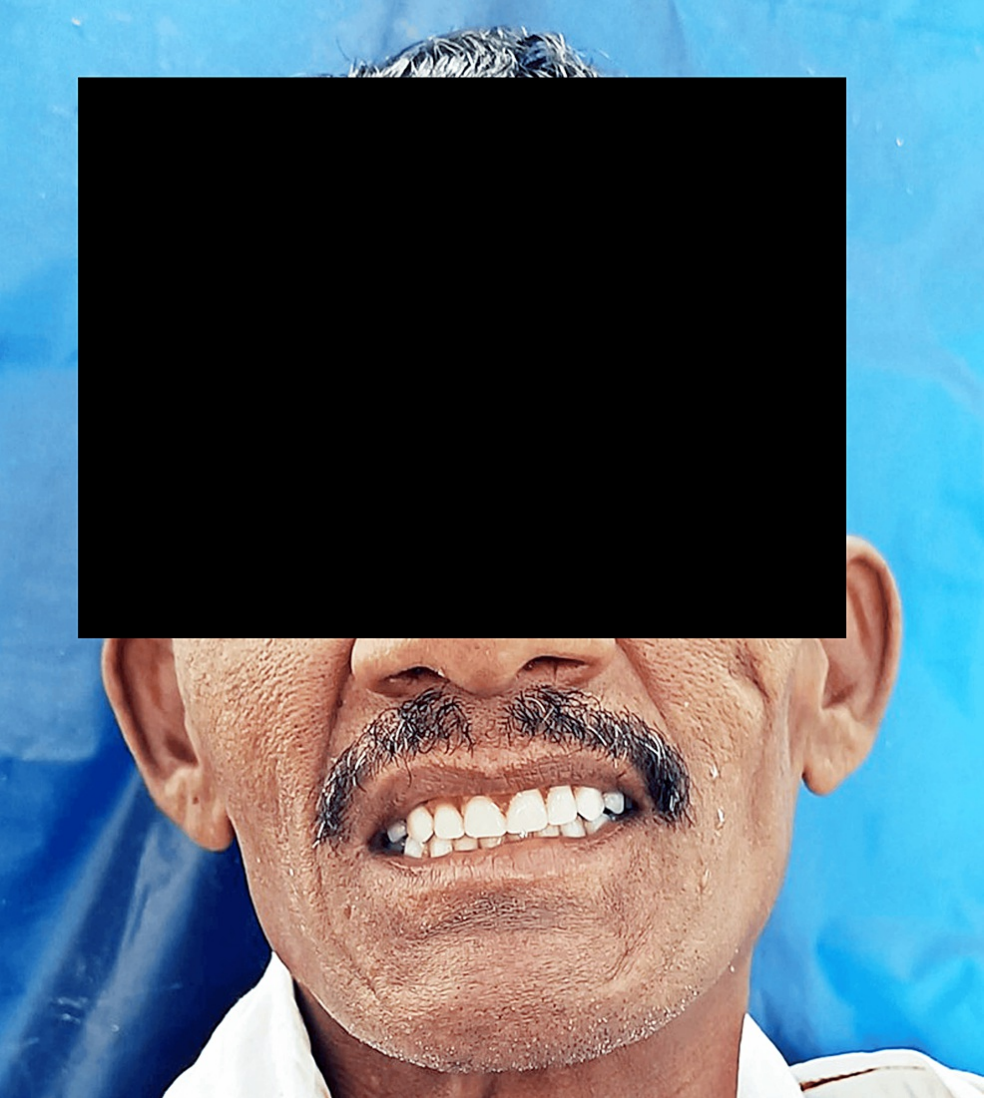
Patient with the final prosthesis and improved aesthetics

Heat-cure polymerizing acrylic resin (Dental Product of India, Mumbai, India) was put into the mold area, and cured following the company's specifications. After the completion of the deflasking, the cured CDs and CPs were removed. Trimming, polishing, and finishing have all been completed. First, the CP was connected to the prosthetic extra-orally to confirm adequate fit. The completed prosthesis with CPs was tested for comfort, function, and aesthetics in the patient's mouth as represented in Figure 5. It was demonstrated to the patient how to connect the plumper to a prosthetic. He was asked to come back every three months to have the prosthesis checked.

## Discussion

Significant changes occur in face tissues as people age, including tissue shrinkage and the accentuation of folds and wrinkles. These alterations have a visible influence on facial aesthetics, frequently resulting in the appearance of sunken cheeks in those who have lost maxillary and mandibular teeth, as well as a decrease in muscle tone and face fat. CPs were used in the treatment plan to address this issue and improve the patient's facial appearance. Traditionally, CPs were made by adding bulk to the maxillary dentures. The downsides of a conventional CP include greater weight, insertion difficulty, muscular weakness, interference with the masseter and buccinator muscle function, and interference with the coronoid process of the lower arch. It is also not appropriate for those with limited mouth opening [5]. To compensate for the extra weight, the removable plumper may be hollowed out using various ways with the use of salt, ice, soap, and vaseline. 

As a result, removable CPs have advanced in favor because of their capacity to minimize prosthesis weight while also allowing for easy assembly and removal. Most importantly, patients can wear the denture with or without the plumpers. Detachable CPs can be made using a variety of ways, including push buttons or press studs, friction locks, stainless steel double die pins [5], customized Co-Cr attachments [6], and magnets. Every connecting approach has its own list of pros and cons [7]. Magnets were used as the attachment method for the CPs in the scenario discussed in this paper. Magnets have a number of advantages, including their tiny and compact size, strong attractive forces, simplicity of cleaning, and simple implantation by both the patient and the dentist. They also reseat themselves mechanically and entail basic laboratory processes for manufacture. The biggest issue with them is their rusting caused by oral secretions. Encapsulated or coated magnets must be employed [8].

It is crucial for a dentist to be able to comprehend and identify the issues that edentulous patients face, choose the best course of action, and reassure them [9]. A complete denture with a detachable CP could offer the appearance of the patient, more youthful with their cheeks well-supported, which serves to boost the patient's self-confidence and provide remarkable stability during varied functions [10]. However, it is critical to recognize the disadvantages of employing magnets. Magnets' corrosion resistance and magnetic characteristics may deteriorate over time. Despite these restrictions, the use of magnets in this context constitutes a realistic and practical approach for boosting face aesthetics and comfort in patients with sunken cheeks and edentulism.

## Conclusions

CPs are easy to make and offer an economical and non-intrusive therapy option for improving facial appearance for people with depressed cheeks. This therapy improves patients' looks as well as their psychological well-being. The alternative method of utilizing magnets, in the presented case, was explored to enhance the patient's facial appearance. The magnetically fastened CP prosthesis efficiently restored the natural curve of the cheeks, considerably increasing both the patient's aesthetics and psychological well-being. Magnetic retention offers significant benefits in situations with hollow cheeks due to its small size and robust attracting forces. Nevertheless, because of their poor long-term endurance in the harsh oral environment, magnets utilized intraorally may require frequent replacement.

## References

[1] Tautin FS (1978). Denture esthetics is more than tooth selection. J Prosthet Dent.

[2] Virdiya NM, Palaskar JN, Wankhade J, Joshi N (2017). Detachable cheek plumpers with different attachments for improving esthetics in a conventional complete denture: a clinical report. J Prosthet Dent.

[3] Abdelbagi NF, Ismail IA, Awadalkreem F, Alhajj MN (2021). Detachable lip and cheek plumper for rehabilitation of facial disfigurement. Case Rep Dent.

[4] Bhushan P, Aras MA, Coutinho I, Rajagopal P, Mysore AR, Kumar S (2019). Customized cheek plumper with friction lock attachment for a completely edentulous patient to enhance esthetics: a clinical report. J Prosthodont.

[5] Pudi S, Kota S, K V G Ch K, Kaladi SR, Gade RR (2019). An innovative technique using a stainless steel double die pin retained cheek plumper in complete denture esthetics: a case report. Cureus.

[6] Kumar D, Rajeshwari CL, Srivastava G, Shetty RM (2019). Hooks retained detachable cheek plumper to enhance aesthetics in a completely edentulous patient - a case report. Int J Oral Health Dent.

[7] Colvenkar S, Pathipaka S, Siva Santosh Babu D, Vijay Kumar K, Prakash R (2022). Enhancing facial esthetics in a complete denture patient having sunken cheeks with a hollow fixed cheek plumper: a case report. Cureus.

[8] Riley MA, Walmsley AD, Harris IR (2001). Magnets in prosthetic dentistry. J Prosthet Dent.

[9] Aggarwal P, Gupta MR, Pawah S, Singh A (2016). An innovative technique to improve complete denture aesthetics using cheek plumper appliance: A case report. Int J Oral Health Med Res.

[10] Bansod AV, Pisulkar SG, Dahihandekar C (2022). Enhancing esthetics in a complete denture patient: optimizing results with different impression techniques. Cureus.

